# Thermolability of mutant MMACHC protein in the vitamin B12-responsive *cblC* disorder

**DOI:** 10.1016/j.ymgme.2010.02.005

**Published:** 2010-05

**Authors:** D.S. Froese, S. Healy, M. McDonald, G. Kochan, U. Oppermann, F.H. Niesen, R.A. Gravel

**Affiliations:** a Department of Biochemistry and Molecular Biology, University of Calgary, Alberta Children’s Hospital Research Institute for Child and Maternal Health, Calgary, Alta., Canada T2N 4N1; b Structural Genomics Consortium, University of Oxford, Old Road Campus Research Building, Roosevelt Drive, Oxford OX3 7DQ, United Kingdom; c Nuffield Department of Orthopedic Surgery, Rheumatology and Musculoskeletal Sciences, Botnar Research Center, Biomedical Research Unit, University of Oxford, Oxford, United Kingdom

**Keywords:** Cobalamin, Differential scanning fluorimetry, *cblC*, Isothermal denaturation, MMACHC, Vitamin B12

## Abstract

Methylmalonic aciduria and homocystinuria, *cblC* type, is the most common inborn error of cellular vitamin B12 metabolism. We previously showed that the protein carrying the mutation responsible for late-onset *cblC* (MMACHC-R161Q), treatable with high dose OHCbl, is able to bind OHCbl with wild-type affinity, leaving undetermined the disease mechanism involved [Froese et al., Mechanism of responsiveness, Mol. Genet. Metab. (2009).]. To assess whether the mutation renders the protein unstable, we investigated the thermostability of the wild-type and mutant MMACHC proteins, either unbound or bound to different cobalamins (Cbl), using differential scanning fluorimetry. We found that MMACHC-wt and MMACHC-R161Q are both very thermolabile proteins in their apo forms, with melting temperatures (*T*_m_) of 39.3 ± 1.0 and 37.1 ± 0.7 °C, respectively; a difference confirmed by unfolding of MMACHC-R161Q but not MMACHC-wt by isothermal denaturation at 35 °C over 120 min. However, with the addition of OHCbl, MMACHC-wt becomes significantly stabilized (Δ*T*_m max_ = 8 °C, half-maximal effective ligand concentration, AC_50_ = 3 μM). We surveyed the effect of different cobalamins on the stabilization of the wild-type protein and found that AdoCbl was the most stabilizing, exerting a maximum increase in *T*_m_ of ∼16 °C, followed by MeCbl at ∼13 °C, each evaluated at 50 μM cofactor. The other cobalamins stabilized in the order (CN)_2_Cbi > OHCbl > CNCbl. Interestingly, the AC_50_’s for AdoCbl, MeCbl, (CN)_2_Cbi and OHCbl were similar and ranged from 1–3 μM, which compares well with the *K*_d_ of 6 μM for OHCbl [Froese et al., Mechanism of responsiveness, Mol. Genet. Metab. (2009).]. Unlike MMACHC-wt, the mutant protein MMACHC-R161Q is only moderately stabilized by OHCbl (Δ*T*_m max_ = 4 °C). The dose–response curve also shows a lower effectivity of OHCbl with respect to stabilization, with an AC_50_ of 7 μM. MMACHC-R161Q showed the same order of stabilization as MMACHC-wt, but each cobalamin stabilized this mutant protein less than its wild-type counterpart. Additionally, MMACHC-R161Q had a higher AC_50_ for each cobalamin form compared to MMACHC-wt. Finally, we show that MMACHC-R161Q is able to support the base-off transition for AdoCbl and CNCbl, indicating this mutant is not blocked in that respect. Taken together, our results suggest that protein stability, as well as propensity for ligand-induced stabilization, contributes to the disease mechanism in late-onset *cblC* disorder. Our results underscore the importance of cofactor stabilization of MMACHC and suggest that even small increases in the concentration of cobalamin complexed with MMACHC may have therapeutic benefit in children with the late-onset, vitamin responsive *cblC* disease.

## Introduction

**Fig. 1:**
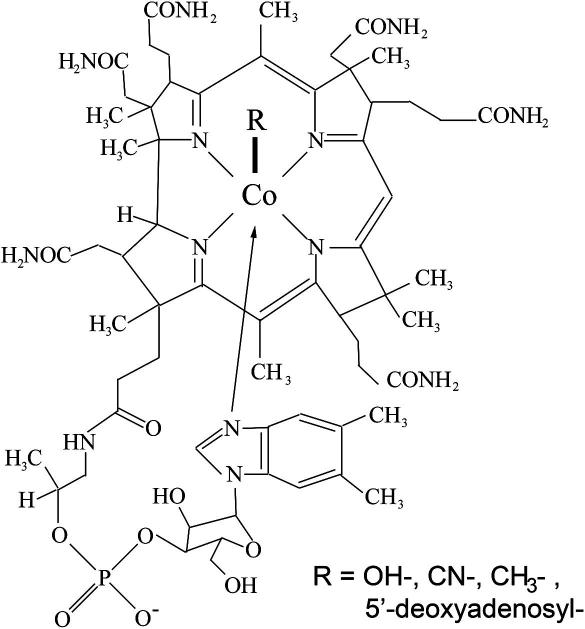
Chemical structure of vitamin B12. The arrow from the DMB group up to the cobalt represents the bond that may exist, making this structure base-on.

Vitamin B12 (cobalamin, Cbl) is a complex organometallic molecule whose structure was determined by Hodgkin et al. [1] in 1956 after being first isolated by Smith [2] and Rickes et al. [3]. The cobalamin core consists of a cobalt atom caged in a corrin ring (Fig. 1). Extending beneath the corrin ring is the 5,6-dimethylbenzimidazole (DMB) base which may be coordinated to the cobalt atom to form the lower or α-axial ligand. When attached, the structure is considered “base-on”; when unattached, “base-off”. If the DMB moiety is not present, the structure is called cobinamide (Cbi). The upper or β-axial ligand varies depending on the modification state of cobalamin (R-group in Fig. 1). Cyanocobalamin (CNCbl) was the form initially crystallized and continues to be the most common commercially available form of cobalamin; however, it does not occur naturally in plants, micro-organisms or animal tissues [4]. Hydroxocobalamin (OHCbl), methylcobalamin (MeCbl) and 5′-deoxyadenosylcobalamin (AdoCbl) are the three forms that have been frequently isolated from mammalian tissues [5] and, for humans, are considered to be the naturally occurring forms.

In mammals, two enzymes use cobalamin as a cofactor. Methionine synthase, a cytosolic enzyme that catalyzes the methylation of homocysteine to form methionine, requires MeCbl as cofactor. The second enzyme, methylmalonyl-CoA mutase, is a mitochondrial enzyme that uses AdoCbl as cofactor. It is the final enzyme in the degradation of branched chain amino acids, odd-chain length fatty acids and cholesterol. Eight complementation groups, *cblA-G* and *mut*, define blocks either in the conversion of cobalamin to cofactor form in the cytosol or mitochondrion or in the activities of the cobalamin-dependent enzymes. Three groups, *cblF*, *cblC* and *cblD* are associated with blocks in lysosomal transport of cobalamin into the cytosol or in early processing steps. Affected patients have combined homocystinuria and methylmalonic aciduria due to a block common to the synthesis of MeCbl and AdoCbl, respectively [5]. Two groups, *cblE* and *cblG*, affect methionine synthase only, while the remaining three groups, *cblA*, *cblB* and *mut*, affect methylmalonyl-CoA mutase activity exclusively. Patients from any of these groups may show a biochemical or clinical response to high-dose vitamin B12 therapy.

Of the complementation groups described, by far the most common disorder is *cblC*, with over 350 patients identified [6]. Clinically, *cblC* is split into two loosely defined groups depending on time of presentation; early-onset for those with symptoms before 1 year of age and late-onset for those any time after [7]. In general, early-onset *cblC* is a much more severe form, characterized by failure to thrive, poor feeding/growth, seizures, hematologic abnormalities and neurological deterioration, with clinical outcome being generally poor despite treatment and metabolic management [7,8]. Late-onset *cblC*, on the other hand, often presents with psychiatric disturbance and neurologic symptoms with or without thrombosis [9]. Importantly, the late-onset form has a much more favorable outcome, including reversal of neurological and psychiatric symptoms if treatment is initiated early enough [9,10].

The gene responsible for *cblC* has been identified and named *MMACHC* [11]. The MMACHC protein has been shown to bind cobalamin and to have, in the presence of a reductase such as methionine synthase reductase or novel reductase 1, the ability to reductively decyanate CNCbl to cob(II)alamin *in vitro* [12], as well as to dealkylate cobalamins containing C2–C6 alkanes, or CN-, adenosyl- or methyl- as the upper axial ligand *in vivo* [13]. These results suggest that MMACHC is responsible for early processing of cobalamin [13] and that binding of MMACHC to cobalamin, in whichever form, is integral to this function. Recently, we used recombinant MMACHC to investigate cobalamin binding with a mutant protein model of early-onset (G147D) and late-onset (R161Q) *cblC*. We found that MMACHC-G147D was unable to bind OHCbl or CNCbl, while MMACHC-R161Q was able to bind OHCbl with wild-type affinity but was impaired in CNCbl binding and had reduced decyanase activity [14]. This work provided a rational explanation for the better response of *cblC* fibroblasts [15] and patients [7,16] to OHCbl compared to CNCbl and demonstrated ablated binding of OHCbl as the basis for functional deficiency in early-onset (G147D) *cblC*. However, since MMACHC-R161Q exhibited normal OHCbl binding, this study failed to explain the disease mechanism in late-onset *cblC* patients. Nonetheless, this study suggested a difference in stability as an intriguing possibility to explain the different disease mechanisms; we observed significant instability of MMACHC-R161Q during recombinant expression as a large fraction of expressed protein was located in the insoluble fraction and a large amount of MMACHC-R161Q protein precipitated upon cleavage of the GST-tag [14].

In the work described here, we investigated the thermostability of the MMACHC-R161Q protein in comparison to wild-type (MMACHC-wt) in the presence or absence of cobalamin as a possible mechanism for dysfunction of the mutant protein. Using differential scanning fluorimetry (DSF), we demonstrate that MMACHC-wt is an exceedingly labile protein, with a melting temperature near physiologic temperature, which, however, is strongly stabilized by binding to cobalamins. We show that MMACHC-R161Q is less stable and that the effect of cobalamin binding on its stability is smaller than for the wild-type protein. Finally, we show that, like MMACHC-wt, MMACHC-R161Q is able to bind CNCbl and AdoCbl in the base-off form. Taken together, our results point to decreased stability and lesser stabilization by cobalamin as contributors to the disease mechanism in the *cblC* disorder.

## Materials and methods

### Materials

OHCbl, CNCbl, AdoCbl, MeCbl and Cbi (as dicyanocobinamide, (CN)_2_Cbi) were purchased from Sigma–Aldrich (Oakville, ON). All other chemicals were reagent grade.

### Plasmid generation and site-directed mutagenesis

The cDNA of MMACHC was obtained from OriGENE (Rockville, MD) cloned into the pCMV6-Entry vector. To transfer the cDNA into pNIC28-Bsa4 (SGC expression vector, GenBank accession EF198106), the primers were TACTTCCAATCCATGGAGCCGAAAGTCGCAGAGC and TATCCACCTTTACTGCTATCAAGGGCCAGGGGATGCAGG where the underlined sequences represent LIC sites. LIC cloning was performed by first digesting the vector with BsaI (NEB; Ipswich, MA) for 2 h at 50 °C, followed by incubating the digested plasmid with insert, made by PCR using the primers above, as well as dGTP (Invitrogen: Burlington, ON), and T4 DNA polymerase (NEB) for 30 min at 22 °C followed by 70 °C for 20 min. Plasmid and insert were then ligated by mixing together for 10 min at room temperature and finally transformed into BL21 DE3 cells following the manufacturer’s instructions. Mutant sequences were generated by site-directed mutagenesis of the wild-type vector using the primers TTGGGGGCTGGTTTGCCATCC**A**AGGGGTAGTGCTGCTGCCAGG and CCTGGCAGCAGCACTACCCCT**T**GGATGGCAAACCAGCCCCCAA for R161Q, where the underlined letters represent the codon to be changed and the letters in bold represent the mutated nucleotides. This was done following the manufacturer’s instructions (Stratagene; La Jolla, CA). All vectors were sequenced to verify the correct insert.

### Protein expression and purification

MMACHC and MMACHC-R161Q in pNIC26-Bsa4 (N-terminal His-tag) were expressed and purified as described by Picaud et al. [17]. Purified protein before treatment with TEV had the N-terminal His-tag intact (His-MMACHC-wt and His-MMACHC-R161Q), while purified protein after TEV treatment had the His-tag removed (MMACHC-wt and MMACHC-R161Q).

### UV–visible absorption spectra

To determine base-on or base-off binding, 5 μM of the appropriate cobalamin or cobinamde in buffer (100 mM Tris, pH 7.0), was incubated in the dark with 400 μl total of 50 μM MMACHC protein (wild-type or R161Q), prepared in the same buffer, or alone, for 5 min at room temperature. In order to retain the high amounts of protein necessary for these experiments, the His-tag (wt and R161Q) or GST-tag (wt protein) was left on. Absorption spectrum measurements were carried out using a MOS-250 spectrometer and recorded using Bio-Kine 32 v4.20 software.

### Differential scanning fluorimetry

DSF was performed and analyzed as described in Niesen et al. [18], with minor modifications. Purified MMACHC proteins were diluted to 0.1 mg/ml (3 μM) in buffer (10 mM HEPES pH 7.5, 0.5 M NaCl, 5% glycerol) with 1:1000 Sypro Orange (Invitrogen) and run in triplicate. The proteins were heated from 5 to 75 °C with a ramp rate of 1 °C/min in a thermocycler (Bio-rad C1000 Thermocycler, CFX96 Real-time system). All graphs were normalized so that minimum fluorescence was set to 0 and maximum fluorescence set to 1. This was done because all cobalamins showed fluorescence quenching at higher concentrations. However, quenching did not affect *T*_m_’s as a control protein (GST) showed no *T*_m_ shift with any cobalamin used. Final graphs and AC_50_’s were generated using Kadeidagraph 4.0 software (Synergy Software, Reading, PA). AC_50_’s were determined by fitting the Δ*T*_m_ vs. ligand concentration curves to the equation Δ*T*_m_ = Δ*T*_m max_[S]/(AC_50 _+ [S]) where Δ*T*_m_ is the change in melting temperature (*T*_m_[protein + ligand] − *T*_m_[protein]), Δ*T*_m max_ is the maximal change in *T*_m_ at saturating (50 μM) ligand concentration and [S] is the ligand concentration.

### Isothermal denaturation (ITD)

ITD was performed as previously described [19] with minor variations. Samples of both proteins prepared similarly as for DSF (at similar concentrations) were kept at constant temperature of 35 °C while monitoring the fluorescence of Sypro orange at intervals of one minute in a thermocycler (Bio-rad C1000 Thermocycler, CFX96 Real-time system) over 120 min (*n* = 12 for each protein).

### Statistics

All experiments were performed at least in triplicate. Error bars shown are ±1 S.D. All statistics were performed using a two-tailed Student’s *T*-test, with significance determined at *p* < 0.05, unless otherwise indicated.

## Results

### MMACHC-R161Q is less stable than the wild-type protein

**Fig. 2:**
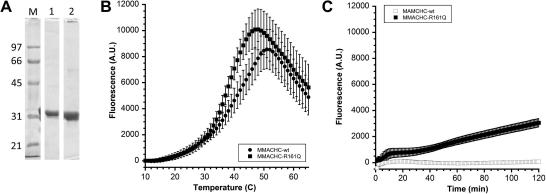
Purification and thermostability of MMACHC-wt and MMACHC-R161Q. (A) SDS–PAGE analysis showing purity of MMACHC-wt and MMACHC-R161Q. Proteins had their His-tags cleaved with TEV and were analyzed by 10% SDS–PAGE with staining by Coomassie blue. M, marker; lane 1, MMACHC-wt; lane 2, MMACHC-R161Q. (B) Thermostability of MMACHC-wt and MMACHC-R161Q. Protein stability was determined by DSF as described in Materials and Methods. Curves are shown as an average (±S.D.) of an *n* ⩾ 9. (C) ITD; course of Sypro orange fluorescence in the presence of MMACHC-wt and MMACHC-R161Q at a constant temperature of 35 °C. Curves shown are an average of *n* = 12. A.U. = arbitrary units.

**Fig. 3:**
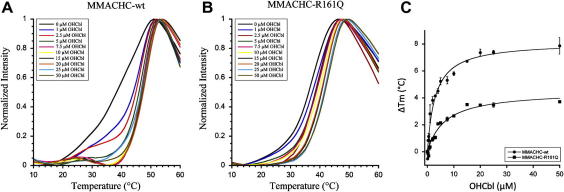
MMACHC-wt and MMACHC-R161Q stabilization by OHCbl. MMACHC-wt (A) and MMACHC-R161Q (B) were incubated with 0, 1, 2.5, 5, 7.5, 10, 15, 20, 25 or 50 μM OHCbl and analyzed by DSF. Each curve is the average of *n* = 3. C. Plot of Δ*T*_m_ vs. OHCbl.

Wild-type (MMACHC-wt) and mutant (MMACHC-R161Q) MMACHC were expressed and purified to >95% purity, with His-tag removed, as judged by SDS–PAGE (Fig. 2A). In order to investigate the thermostability of MMACHC we used DSF, a method that measures protein stability and ligand-induced changes in stability [20,21]. DSF uses an environmentally sensitive dye, such as Sypro orange, to monitor protein-unfolding caused by heat denaturation. As the protein unfolds, the dye, which has a higher fluorescence quantum yield in nonpolar environments than in aqueous media, binds to hydrophobic regions of the protein, causing a large increase in fluorescence [22,23]. Protein stability is expressed as the melting point of the protein (*T*_m_), that is, the transition midpoint of the fluorescence curve, corresponding to the temperature at which half of the protein molecules are folded and half are unfolded [22]. By this method, we found that the melting temperature for MMACHC-wt was only slightly higher than physiological temperature (*T*_m_ = 39.3 ± 1.0 °C). Even lower was the stability of MMACHC-R161Q (*T*_m_ = 37.1 ± 0.7 °C) (Fig. 2B). The difference in *T*_m_ was small but nonetheless significant (*p* < 0.01). A second difference between the wild-type and mutant proteins was the shape of the unfolding curves, with the unfolding of the mutant protein showing high cooperativity indicating a two-state unfolding mechanism while the unfolding of the wild-type protein stretched over a larger temperature range and comprised two phases (Fig. 2B; also Fig. 3A at 0 μM OHCbl).

We performed isothermal calorimetry (ITD) to investigate further the difference in stability between the two proteins. ITD has been described as being more sensitive than DSF for detecting small changes in protein stability [19]. ITD measures the unfolding of proteins at a constant temperature, typically chosen as a few degrees below the *T*_m_, using a similar procedure as DSF including the use of Sypro orange as the reporter dye. Performed at 35 °C, ITD showed the unfolding of MMACHC-R161Q from the start of the experiment, indicated by an increase of the fluorescence intensity (Fig. 2C). After 120 min the intensity reached approximately half of the amplitude seen for unfolding with DSF (Fig. 2B). The MMACHC-wt, in contrast, did not unfold over the duration of the experiment. This difference cannot be explained with the relatively small difference in the *T*_m_. If ITD is performed at a temperature that falls within the range of the unfolding transition a gradual increase in the proportion of unfolded protein molecules, i.e. a constant increase in fluorescence intensity, would still be expected even at temperatures further away from the *T*_m_ (18).

### Differential stabilization of MAMCHC proteins by cobalamins

**Fig. 4:**
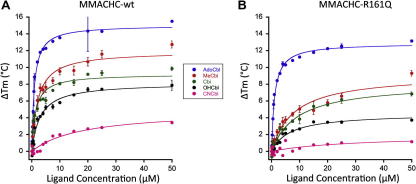
Stabilization of MMACHC-wt and MMACHC-R161Q by binding different forms of cobalamin and cobinamide. MMACHC-wt (A) and MMACHC-R161Q (B) were incubated with 0, 0.1, 0.25, 0.5, 0.75, 1, 2, 3, 4, 5, 7.5, 10, 15, 20, 25 or 50 μM of AdoCbl, MeCbl, OHCbl, (CN)_2_Cbi and CNCbl and analyzed by DSF in triplicate. Each data point represents the average ± 1 S.D. The accompanying table (Table 1) displays the half-maximal effect concentrations, AC_50_, as well as the observed maximal stabilization Δ*T*_m max_, calculated from the difference between the *T*_m_ at 50 μM ligand and the *T*_m_ at 0 μM ligand for the different protein:ligand pairs.

To determine whether binding of cobalamins to the two proteins results in an increase in protein stability, we incubated MMACHC-wt and MMACHC-R161Q with increasing concentrations of OHCbl. Dependent on the ligand concentration, both proteins showed very strong increases in *T*_m_ (up to ∼8 °C for the wild-type), indicating that they were stabilized by the binding to OHCbl (Fig. 3A,B). However, MMACHC-wt showed stronger stabilization by OHCbl than did MMACHC-R161Q, by approximately 4 °C (Fig. 3C). Moreover, the sensitivity of MMACHC-wt for stabilization by OHCbl was greater than obtained for MMACHC-R161Q, shown by a lower concentration of ligand that was necessary to achieve half-maximal stabilization (AC_50_, Fig. 3C; Table 1). In order to stabilize MMACHC-R161Q half-maximally, 6.6 μM of OHCbl was necessary, while only 3.0 μM OHCbl was sufficient to achieve the half-maximal stabilization of MMACHC-wt (Table 1). Interestingly, the values of AC_50_ for MMACHC-wt and MMACHC-R161Q with OHCbl correspond well to published *K*_d_’s (MMACHC-wt AC_50_ = 3.0 ± 0.4, *K*_d_ = 5.7 ± 2.2; MMACHC-R161Q AC_50_ = 6.6 ± 1.2, *K*_d_ = 3.8 ± 2.0) (Table 1;[14]). Strikingly, we also observed a difference in the effect of the ligand on the shape of the curves between MMACHC-wt and MMACHC-R161Q. The previously described (see above) strong cooperativity of unfolding of the mutant protein remained at all ligand concentrations and the transitions were horizontally shifted towards higher temperatures (Fig. 3B). However, for the wild-type protein the cooperativity increased with the concentration of ligand and two-state unfolding became apparent at saturating concentrations of OHCbl. Since MMACHC has been shown to bind different cobalamins [12], we determined which form might result in the greatest stabilization of MMACHC. We found that MMACHC-wt was stabilized most by the two cofactor forms of cobalamin, AdoCbl and then MeCbl (Fig. 4A; Table 1). It was stabilized to a lesser degree by OHCbl and dicyanocobinamide ((CN)_2_Cbi) and least by CNCbl (Fig. 4A; Table 1). Interestingly, MMACHC-R161Q showed the exact same pattern of stabilization, except that all cobalamins stabilized MMACHC-R161Q to a lesser extent than MMACHC-wt (Fig. 4B; Table 1). Using the curves in Fig. 4A and B we determined the AC_50_ for each cobalamin form. We found that, similar to our observation with OHCbl, MMACHC-wt was more sensitive to stabilization by the other cobalamins than MMACHC-R161Q, shown by the generally lower values for AC_50_ of the mutant protein (Table 1). We do note, however, that the AC_50_ of MMACHC-R161Q for CNCbl had a larger standard deviation than concentration (Table 1), most likely due to the extremely small stabilization gain with this form of cobalamin and the poor fit of the curve.

### Binding conformation of different cobalamins

**Fig. 5:**
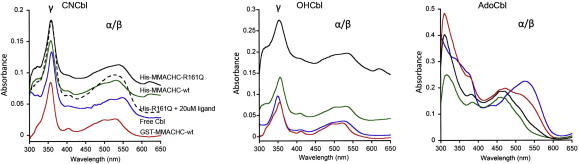
Conformation of cobalamins when bound to MMACHC-wt and MMACHC-R161Q at pH 7.0. The UV–visible absorption spectra are shown for CNCbl (left-panel), OHCbl (central-panel) and AdoCbl (right-panel) either as free or bound to GST-MMACHC-wt, His- MMACHC-wt or His-MMACHC-R161Q. Sample colours are as follows: black, His-MMACHC-R161Q; green, His-MMACHC-wt; blue, free Cbl; red, GST-MMACHC-wt. The dashed black line in the CNCbl panel represents MMACHC-R161Q with 20 μM ligand. The peak positions and consequent judgement of base-on/off conformation of the cobalamin are summarized in the accompanying table (Table 2).

Previously, we demonstrated that GST-MMACHC-wt binds CNCbl in the base-off state [14]. Additionally, His-MMACHC-wt had also been shown to bind AdoCbl and MeCbl base-off [12]. In order to determine whether the R161Q mutation interferes with the ability of MMACHC to support this transition, we examined the UV–visible absorption spectra for the proteins bound to AdoCbl, as an example of an alkylcobalamin, as well as for the proteins bound to CNCbl and OHCbl. Each cobalamin was added at a concentration of 5–50 μM protein (Fig. 5). To determine whether the cobalamin was base-on or base-off, in the case of CNCbl both base-on and base-off spectra were determined for the free vitamin, as previously reported [12,14]. For OHCbl and AdoCbl, only base-on spectra were determined for the free molecule and the complex with MMACHC judged to be base-off if the peak at ∼530 nm (α/β) shifted significantly towards 500 nm, as that has been shown to indicate the substitution of the DMB by H_2_O in the lower axial position of the cobalt [24–26]. Confirming our earlier data, GST-tagged MMACHC-wt bound CNCbl in the base-off state (Table 2). The fact that the spectra were similar for His-tagged MMACHC-wt shows that the nature of the tag is unlikely to interfere with cobalamin binding. Both MMACHC-wt and MMACHC-R161Q bound AdoCbl in the base-off state (Fig. 5; Table 2). However, for CNCbl, the spectrum of cobalamin binding to MMACHC-R161Q was shifted incompletely towards full base-off binding compared to MMACHC-wt (Fig. 5, black solid line; Table 2). Only at a higher concentration of CNCbl (20 μM, at the *K*_d_ obtained for the mutant protein [14]) was full base-off binding observed (Fig. 5, black dotted line; Table 2). Finally, neither MMACHC-wt nor MMACHC-R161Q modified OHCbl binding, which retained the base-on profile regardless of the MMACHC species present (Fig. 5; Table 2).

## Discussion

We examined the thermolability of a mutant form of MMACHC that is responsive to OHCbl but not to CNCbl in patients. Our previous finding that recombinant MMACHC-R161Q binds OHCbl with wild-type affinity provided a facile explanation for why patients respond clinically to OHCbl. We had shown that the mutant protein binds CNCbl poorly and that it’s reduced decyanase activity correlated with the reduced binding, but it left perplexing why patients with at least one R161Q allele have disease at all. Here, we addressed the thermostability of wild-type and R161Q mutant protein in the presence or absence of cobalamins and assessed the configuration taken on by cobalamins when bound to mutant protein. Our results demonstrated a reduced propensity of the mutant protein to be stabilized by cobalamin binding compared to the wild-type protein, suggesting that impaired B12-mediated stabilization is key to the disease pathology associated with this mutation and providing a rationale for why an increase in B12 concentration is therapeutic.

To study MMACHC-wt and MMACHC-R161Q thermostability we used DSF. This method, which monitors the heat-induced unfolding of proteins in the presence of a fluorescent dye [21,27], allowed us to conduct an in depth survey of the effect of cobalamins on protein stabilization. Unexpectedly, we found that MMACHC-wt itself is a very thermolabile protein, with a *T*_m_ of 39.3 ± 1.0 °C. This is a lower *T*_m_ than obtained for 39 out of 42 human proteins (93%) investigated by Vedadi et al. [28] using the same method. Interestingly, the R161Q mutation caused the protein to have even lower stability, with a reduction in *T*_m_ to 37.1 ± 0.7 °C. We also observed a difference in the mode of unfolding between the proteins. Unfolding of most proteins follows a two-state mechanism, i.e., the native state transforms into the unfolded state without any intermediary step. In such cases, the graphical representation of the number of unfolded proteins versus the reaction coordinate (here: temperature axis) has a sigmoid shape. Any deviation from the sigmoid observed in a transition curve indicates the involvement of additional, intermediate states in the unfolding process. Interestingly, we found that MMACHC-R161Q unfolding more closely followed a two-state mechanism than the wild-type protein. Strong support for the interpretation towards a deviation from a two-state mechanism is given by the results from the isothermal denaturation (ITD) experiments. Commonly, ITD experiments are performed at a temperature near the onset of unfolding, obtained from DSF curves. Under such conditions, most proteins show a progressive increase in the fluorescence intensity over time [19]. For the MMACHC proteins, the stability over time was assessed at near-physiological temperature (35 °C), i.e., at much higher temperature than the onset of unfolding (∼20 °C). It is therefore remarkable that the wild-type protein did not unfold over the course of two hours, which seems to indicate a high energy-barrier for the transition from either the native to the intermediate state, or from the intermediate state to the fully unfolded state that is not overcome at the incubation temperature. In contrast, the mutant protein showed a progressive increase in fluorescence indicative of increasing exposure of hydrophobic sites to the indicator dye following a lag phase of approximately 20 min. We speculate that due to the mutation the described energy-barrier is decreased in comparison to the wild-type protein. The observation of a lag phase during ITD of the mutant protein seems to argue that the energy-barrier difference between the two proteins concerns the first of the two transitions, i.e., the transition from the native to the intermediate state. Because, in the mutant, the energy that is required to reach the intermediate state is lower than in the wild-type protein the majority of the protein molecules quickly collate in this state, therefore ‘becoming available’ to undergo the second transition and unfold completely. In contrast, for the wild-type protein at equilibrium the native state is strongly favored over the intermediate state at the near-physiological temperature of the ITD experiment, thus reducing the likelihood of complete unfolding to near-zero. Taken together, the data from DSF and ITD show a strong difference in stability between the wild-type and mutant proteins and indicate a much shortened half-life for the mutant protein under physiological conditions.

Our results show that binding of cobalamins to the wild-type and mutant proteins induces a dramatic and concentration-dependent increase in their thermal stability. With the exception of CNCbl, the maximum effect on the stability of all cobalamins shifted the *T*_m_ of both proteins above the physiological temperature. However, we observed significant differences between the proteins with respect to both the extent of stabilization caused by the cobalamins as well as the effectivity of the effect (indicated by the concentration of ligand that is necessary to achieve half-maximal stabilization, AC_50_). In general, the propensity of MMACHC-wt to be stabilized by cobalamins is greater than that of MMACHC-R161Q. For example, the mutant protein is only half as much stabilized by OHCbl and is also less sensitive to OHCbl than the wild-type protein (AC_50_ = 6.6 μM versus 3.0 μM, respectively). Therefore, our results suggest that, at the low levels of OHCbl expected to exist intracellularly [29], wild-type MMACHC is significantly better stabilized by binding to OHCbl than the R161Q mutant form, suggesting that the mutant protein may be subject to a more rapid turnover compared to wild-type MMACHC. It will be interesting to examine the protein in cells *in situ* or by Western blot analysis following growth in high versus absent vitamin B12, when MMACHC-specific antibodies become available. It is important to note, however, that patients with the R161Q mutation have been seen to respond well to OHCbl therapy [30]. Therefore, even a small increase in the intracellular OHCbl concentration obtained with high dose OHCbl therapy may be enough to stabilize the mutant protein sufficiently to perform its function.

The present study demonstrates large differences among the cobalamins and cobinamide with respect to their effect on the stability of MMACHC. The two alkyl cobalamin cofactors, AdoCbl and MeCbl, were most effective in stabilizing the protein, (CN)_2_Cbi and OHCbl both had intermediate effects, and CNCbl stabilized the protein only weakly. MMACHC containing the R161Q mutation showed a similar pattern of *T*_m_ profiles as the wild-type protein, but the extent of stabilization of all cobalamins was generally smaller than for the wild-type (e.g., reducing Δ*T*_m_ for CNCbl to only 1 °C). Interestingly, this pattern in the stabilization effects of cobalamins correlates well with the observed binding conformations: AdoCbl and MeCbl bound MMACHC in the base-off configuration and did so with the mutant protein as well. CNCbl bound wild-type protein base-off but showed reduced base-off binding with the mutant, with the UV–visible spectrum only shifted to the base-off form at the *K*_d_, suggesting that this cobalamin form is more difficult to switch to the base-off form. OHCbl, by contrast, appeared to be retained completely base-on. These differences likely relate to the influence of the upper axial ligand on the strength of the Co-DMB bond in the lower axial position. The adenosyl (Ado) moiety weakens the Co-DMB bond strength the most, lengthening it to 2.237 Å, while the methyl (Me) group has a lesser effect (to 2.162 Å), followed by CN (2.041 Å) and OH (1.195 Å) [31–33]. Taken together, these data give evidence that the DMB moiety, presumably in base-off configuration, strongly contributes to the stability of MMACHC, with the alkyl cobalamins, which are easily rendered base-off, the most stabilizing, OHCbl and (CN)_2_Cbi which have a base-on or no DMB, respectively, resulting in intermediate stability and CNCbl, which is held incompletely base-off, the least stabilizing.

A surprising outcome is the effective dose of vitamin required to stabilize the wild-type versus mutant protein, represented by the AC_50_. Binding of both OHCbl and MeCbl to R161Q was significantly (2–3 times) less effective than to the wild-type protein. CNCbl, which binds very poorly, did not give a reliable AC_50_ with either protein. These data, therefore, indicate that the mutation causes a disruption of the interaction with the DMB moiety. The observation of a sixfold elevated AC_50_ combined with reduced stabilization for (CN)_2_Cbi where DMB is absent supports this interpretation. In contrast, AdoCbl binding was least disrupted in the mutant protein, i.e., we determined similar AC_50_ values for wt and R161Q. It is possible that the increased opportunity for hydrogen bonding to the larger, more complex Ado group contributes to a more stable interaction with the MMACHC protein. In the R161Q mutant, these putative additional bonding interactions with the Ado group may compensate for the weaker binding below the corrin ring.

It is interesting to relate these data to possible treatments or phenotype outcomes, especially with consideration of the cofactor forms. However, two key points brought up by the literature must be kept in mind. (1) B12 delivered to the cell in either the MeCbl or AdoCbl form is converted to a cobalamin intermediate inside the cell shortly after entry [34,35], most likely cob(II)alamin, and most likely by MMACHC [13,36]. (2) B12 is directed to the mitochondrial or cytosolic pathways after processing by MMACHC, possibly with involvement of the MMADHC protein [37]. Therefore, with cobalamin cofactors processed to a common intermediate followed by downstream sorting, we would not expect either pathway to benefit disproportionately from MeCbl or AdoCbl therapy. Indeed, in clinical testing, MeCbl has been used as an alternative therapy to OHCbl in *cblC* [38] and *cblE* patients [39] and AdoCbl therapy has been attempted multiple times with *cblB* patients [35,40–41], either to no effect or to a mild to moderate effect confounded by the addition of other treatments given concurrently (e.g. folate). This data notwithstanding, the only treatment that might warrant additional consideration may be AdoCbl therapy in *cblC* patients. Kim et al. [36] showed that dealkylation of AdoCbl proceeds slowly compared to MeCbl, while we have previously shown that the R161Q mutation impairs decyanation of CNCbl [14], suggesting that the MMACHC-R161Q protein may be very slow in dealkylation of AdoCbl. These results, in combination with the results presented here that demonstrate increased protein stabilization by AdoCbl, suggest that AdoCbl, given in high dose as a treatment, may persist in the cell long enough to grant a small stabilization of mutant MMACHC. One needs to bear in mind, however, that the actual intracellular concentration of cobalamin is very low (0.03–0.6 μM [29]) and circulating cobalamins rise only approximately 10-fold with therapy [5,42] and therefore probably less so intracellularly, meaning that the gain in stability may be very small. Still, titrating the effect of AdoCbl versus OHCbl as supplements in *cblC* cell cultures may be useful to determine if AdoCbl confers a greater stabilization on mutant protein than OHCbl within the cellular milieu. It might have application to *cblC* patients, perhaps with other mutations, who do not respond well to OHCbl.

While our data show that a strong difference in thermostability exists between wild-type MMACHC and the R161Q protein, it is unclear to what extent the thermostability data reported here can be extrapolated to the intracellular environment. The near physiologic *T*_m_ of the wild-type protein was unexpected and, intuitively, would unlikely apply *in vivo*. The MMACHC protein is expected to act as an intracellular shuttle of cobalamin [43], possibly docking with the cobalamin efflux protein, LMBRD1 [44] for delivery of the vitamin to the cytosol, and with the MMADHC protein, proposed to be responsible for sorting cobalamin between the cytosol and mitochondrial compartments [45]. While such interactions might stabilize the protein intracellularly, there is no basis for speculating at this time that in shuttling between such sites there might be other interacting proteins, such as conferring a chaperone role, or perhaps simply a more stabilizing environment. An example of a large difference in *in vitro* and *in vivo* thermostability is illustrated by firefly luciferase used as a reporter gene in cellular expression experiments. While its half-life is as little as 3 min at 37 °C *in vitro*, it increases to 49 min at 37 °C when expressed in mammalian cell culture [46]. Thermostable mutant luciferase similarly showed an enhanced half-life when expressed in cells, suggesting that the cellular environment was responsible for the enhanced half-life *in vivo*. The authors speculated that the basis of intracellular protection was chaperone activity, since steady-state luciferase levels were reduced by geldanamycin, an inhibitor of Hsp90.

While it would be premature to extrapolate the absolute melting temperatures to the intracellular environment, the present data provide strong evidence that a lower stability of the mutant protein relative to wild-type MMACHC combined with impaired stabilization on B12 binding constitute the basis for the disease mechanism in patients with the R161Q mutation. Importantly, our findings also provide a rationale for the successful treatment of patients with high dose OHCbl. We suggest that a failure to reach a stabilized conformation state on binding OHCbl can be compensated for by increasing the intracellular concentration of the vitamin. Our results implicate decreased protein stability as the defect in R161Q *cblC* disease and highlight the importance of high dose OHCbl, and possibly in some cases AdoCbl, in the treatment of the *cblC* disorder.

## Figures and Tables

**Table 1:** Apparent AC_50_ values of MMACHC-wt and MMACHC-R161Q proteins.

	MMACHC-wt		MMACHC-R161Q	
	AC_50_ (μM)	Δ*T*_m max_ (°C)	AC_50_ (μM)	Δ*T*_m max_ (°C)
AdoCbl	0.9 ± 0.1	15.5 ± 0.1	1.1 ± 0.1	13.2 ± 0.1
MeCbl	2.1 ± 0.3	12.7 ± 0.4	8.0 ± 1.6	9.3 ± 0.3
Cbi	1.3 ± 0.2	9.8 ± 0.2	9.1 ± 1.6	6.8 ± 0.2
OHCbl	3.0 ± 0.4	7.9 ± 0.6	6.6 ± 1.2	3.7 ± 0.1
CNCbl	18.1 ± 6.3	3.4 ± 0.6	29.2 ± 41.4	1.1 ± 0.6

**Table 2:** UV–visible absorption spectra peaks and judgment regarding presence of base-on or off conformation.

	CNCbl			OHCbl			AdoCbl		
	γ-Peak	α/β-Peak	Base-on/off	γ-Peak	α/β-Peak	Base-on/off	γ-Peak	α/β-Peak	Base-on/off
Base-on	360	551		350	525		–	524	
Base-off	355	529					–	460	
GST-MMACHC-wt	357	530	Off	355	522	On	–	472	Off
His-MMACHC-wt	358	531	Off	356	532	On	–	460	Off
His-MMACHC-R161Q	359	540	On/Off	354	535	On	–	461	Off
His-MMACHC-R161Q (20 μM CNCbl)	359	531	Off						
